# RNA binding protein HuD mediates the crosstalk between β cells and islet endothelial cells by the regulation of Endostatin and Serpin E1 expression

**DOI:** 10.1038/s41419-022-05465-6

**Published:** 2022-12-05

**Authors:** Myeongwoo Jung, Seungyeon Ryu, Chongtae Kim, Seongho Cha, Hoin Kang, Eunbyul Ji, Youlim Hong, Youngjoon Lee, Sukyoung Han, Seung Min Jeong, Wook Kim, Eun Kyung Lee

**Affiliations:** 1 Department of Biomedicine & Health Sciences, Institute for Aging and Metabolic Diseases, College of Medicine, The Catholic University of Korea, Seoul, 06591 South Korea; 2 Department of Biochemistry, Institute for Aging and Metabolic Diseases, College of Medicine, The Catholic University of Korea, Seoul, 06591 South Korea; 3 Catholic Institute for Visual Science, Institute for Aging and Metabolic Diseases, College of Medicine, The Catholic University of Korea, Seoul, 06591 South Korea; 4 Institute for Aging and Metabolic Diseases, College of Medicine, The Catholic University of Korea, Seoul, 06591 South Korea; 5 Department of Molecular Science & Technology, Ajou University, Suwon, 16499 South Korea

**Keywords:** RNA quality control, Mechanisms of disease, Type 2 diabetes, RNA

## Abstract

RNA binding protein HuD plays essential roles in gene expression by regulating RNA metabolism, and its dysregulation is involved in the pathogenesis of several diseases, including tumors, neurodegenerative diseases, and diabetes. Here, we explored HuD-mediated differential expression of secretory proteins in mouse insulinoma βTC6 cells using a cytokine array. Endostatin and Serpin E1 that play anti-angiogenic roles were identified as differentially expressed proteins by HuD. HuD knockdown increased the expression of α chain of collagen XVIII (Col18a1), a precursor form of endostatin, and Serpin E1 by associating with the 3′-untranslated regions (UTRs) of *Col18a1* and *Serpin E1* mRNAs. Reporter analysis revealed that HuD knockdown increased the translation of EGFP reporters containing 3′UTRs of *Col18a1* and *Serpin E1* mRNAs, which suggests the role of HuD as a translational repressor. Co-cultures of βTC6 cells and pancreatic islet endothelial MS1 cells were used to assess the crosstalk between β cells and islet endothelial cells, and the results showed that HuD downregulation in βTC6 cells inhibited the growth and migration of MS1 cells. Ectopic expression of HuD decreased Col18a1 and Serpin E1 expression, while increasing the markers of islet vascular cells in the pancreas of *db*/*db* mice. Taken together, these results suggest that HuD has the potential to regulate the crosstalk between β cells and islet endothelial cells by regulating Endostatin and Serpin E1 expression, thereby contributing to the maintenance of homeostasis in the islet microenvironment.

## Introduction

HuD, a member of the Hu family RNA binding proteins, plays an important role in gene expression by regulating RNA metabolism in the brain and certain types of endocrine cells, including pancreatic α and β cells, and small cells lung carcinoma (SCLC) [1–3]. HuD is essential for normal brain function and its aberrant expression is associated with the pathogenesis of several diseases, such as Alzheimer’s disease, amyotrophic lateral sclerosis, and schizophrenia [2, 4, 5]. HuD also executes diverse roles in pancreatic β cells and its differential expression leading to β cell dysfunction has been reported in pancreatic neuroendocrine tumors and type 2 diabetes mellitus [6, 7]. HuD regulates various cellular processes by mediating the turnover or translation of target mRNAs involved in cell growth, death, differentiation, neuronal plasticity, autophagy, metabolism, mitochondrial dynamics, and cellular senescence [8–13]. In addition, HuD has the potential to determine the levels of secretory proteins, including insulin, glucagon, and CCL2, by regulating their biosynthesis at the post-transcriptional level [14–16]. Several studies have been made to identify the molecular targets of HuD and to elucidate HuD-mediated gene regulation [4, 17, 18]; and these efforts will help to understand the pathophysiological roles of HuD in health and diseases.

Pancreatic β cells live in a complex and highly integrated islet microenvironment where they interact with various types of components, including endocrine cells (α, β, and δ cells), vascular cells (endothelial cells and pericytes), immune cells (macrophages and monocytes), neurons, and extracellular matrix molecules [19–21]. Crosstalk between β cells and other components in the islet microenvironment is critical for the maintenance of intact integrity and function of β cells that govern systemic glucose homeostasis. Islet vascular cells, including endothelial cells and pericytes, form the intra-islet microvasculature and affect β cell proliferation, differentiation, and insulin secretion [22–26]. Immune cells play a role in the development of β cell dysfunction by mediating inflammation within islet [27–29]. β cells also communicate with vascular cells and macrophages to ensure their proper functions in the islet [21, 26, 29]. Several reports have shown that disruption of the islet microenvironment resulting from aberrant communications between cells in the islet is associated with several pathological conditions, such as pancreatitis, diabetes, and cancers [21]; however, the detailed mechanisms need to be further elucidated.

To explore the role of HuD in the maintenance of the islet microenvironment, we investigated the differential expression of secretory proteins in pancreatic β cells using mouse insulinoma βTC6 cells and their effects on crosstalk with endothelial cells. We identified Endostatin and Serpin E1 that have anti-angiogenic effects as novel targets of HuD and further showed that HuD negatively regulates their expression and secretion, by binding to their 3′-untranslated regions (UTRs). The conditioned medium of HuD-downregulated βTC6 cells reduced the proliferation and migration of islet endothelial MS1 cells. Taken together, these results suggest that HuD has the potential to regulate the crosstalk between β cells and MS1 cells by regulating the expression of Endostatin and Serpin E1, thereby contributing to the maintenance of homeostasis in the islet microenvironment.

## Results

### Identification of secretory proteins differentially expressed in mouse insulinoma βTC6 cells

**Fig. 1:**
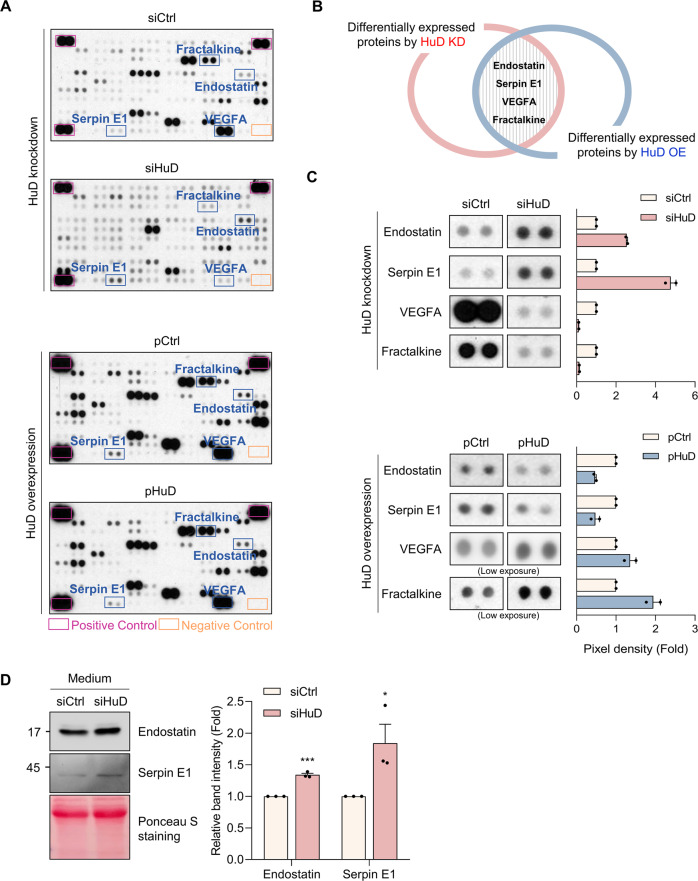
Analysis of differentially expressed proteins secreted by βTC6 cells. **A** After transfection of βTC6 cells with siRNAs or plasmids, the conditioned medium was collected and analyzed using the Proteome Profiler Mouse XL cytokine array. Differentially expressed proteins were marked with rectangles. **B** Comparative analysis identified four proteins, including Endostatin, Serpin E1, VEGFA, and Fractalkine, as the differentially expressed proteins by HuD. **C** Relative levels of four proteins were analyzed using the Image J program. **D** After transfection of βTC6 cells with siRNAs, the levels of Endostatin and Serpin E1 in the conditioned medium were assessed by western blotting analysis. Ponceau S staining was used for total protein normalization. Images are representative and graphs represent mean ± SEM of three independent experiments. The statistical significance of the data was analyzed via Student’s t-test; *p < 0.05; ***p < 0.001.

HuD functions as a pivotal regulator of gene expression in neurons and pancreatic β cells [1, 2]. Our previous researches have shown that HuD regulates the production of secretory proteins, such as proinsulin [16], proglucagon [15], and C–C motif ligand 2 (CCL2) [14], leading us to hypothesize that HuD regulates the expression of secretory proteins. To test this hypothesis, we investigated the relative levels of proteins in the conditioned medium of mouse insulinoma βTC6 cells using the Proteome Profiler Mouse XL Cytokine Array Kit (R&D Systems, Inc.). HuD knockdown or overexpression affected the levels of several proteins in the conditioned medium of βTC6 cells, as shown in Fig. 1A. Comparative analysis identified several proteins involved in the regulation of angiogenesis, including Endostatin, Serpin E1, VEGFA, and Fractalkine, as differentially expressed proteins according to relative HuD levels (Fig. 1B, C). HuD knockdown increased the levels of Endostatin and Serpin E1 that have anti-angiogenic roles, while decreasing the expression of VEGFA and Fractalkine that promote angiogenesis. Ectopic expression of HuD reversed the change in expression of those proteins. Additional western blotting analysis was performed to verify whether HuD directly affects the differential expression of angiogenesis-related factors, and the results revealed that HuD knockdown increased the levels of Endostatin and Serpin E1, but not VEGFA and Fractalkine (Supplementary Fig. S1), in the conditioned medium of βTC6 cells (Fig. 1D). These results suggest that HuD plays a role in regulating the levels of secretory proteins that may affect cell-to-cell communications with nearby cells.

### Alteration in the islet microenvironment in HuD KO mice

**Fig. 2:**
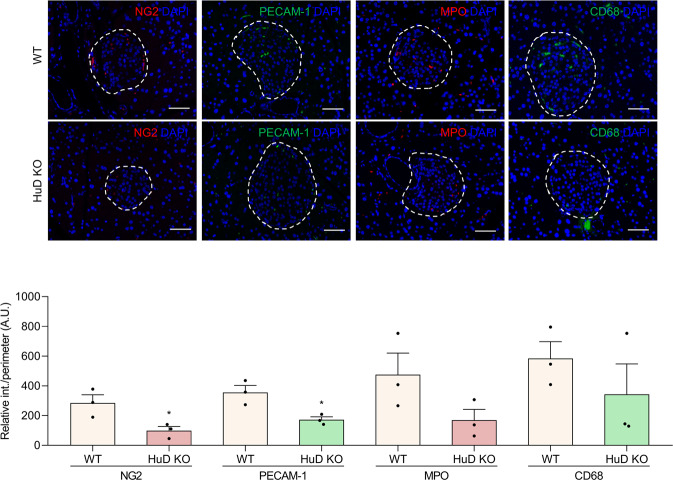
Alterations of cell marker proteins in the pancreatic islet of HuD KO mice. Several marker proteins of pericytes (NG2), endothelial cells (PECAM-1), neutrophils (MPO), and macrophages (CD68) in the pancreatic tissues were analyzed by immunofluorescence microscopy. The nuclei were stained with DAPI solution. Fluorescent signals between wildtype (WT) and HuD knockout (KO) mice were quantified using the Image J program. Data indicate the mean ± SEM and images are representative. Scale bar, 50 μm. The statistical significance of the data was analyzed via Student’s *t* test; *p < 0.05.

The pancreatic islet forms a complex microenvironment that is composed of several types of cells and precise crosstalk between cells is essential for its homeostasis. To determine whether loss of HuD affects the islet microenvironment, the cells in the islet of HuD knockout (KO) mice were analyzed by immunofluorescence microscopy using several cell type-specific markers, including NG2 (pericytes), PECAM-1 (endothelial cells), MPO (neutrophils), and CD68 (macrophages). Signals from NG2-positive cells and PECAM-1-positive cells were downregulated in the islet of the HuD KO mice, compared to wildtype control mice (Fig. 2). However, signals from MPO-positive or CD68-positive cells were moderately, but not significantly altered in the islet of HuD KO mice. These findings indicate that the number of vascular cells, including endothelial cells and pericytes, in the islet microenvironment of HuD KO mice was reduced, implying that HuD may play a role in the regulation of vascular cells in the islet.

### HuD-mediated regulation of Endostatin and Serpin E1

**Fig. 3:**
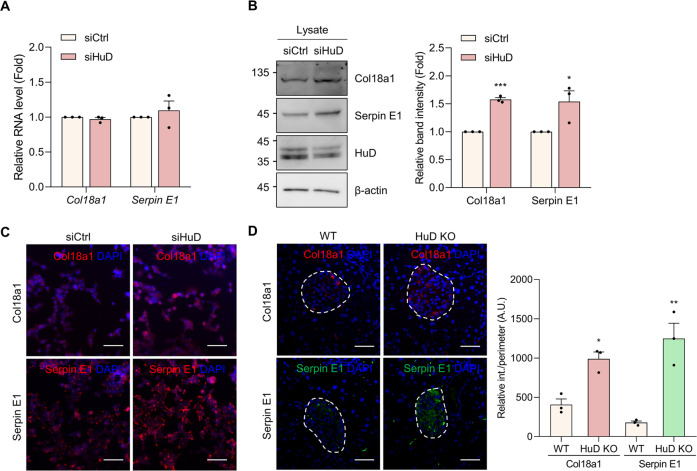
Augmented expression of Col18a1/Endostatin and Serpin E1 by HuD knockdown. **A**, **B** After transfection of βTC6 cells with siRNAs, the expression of Col18a1 and Serpin E1 was assessed by RT-qPCR (**A**) and western blotting analysis (**B**). *Gapdh* mRNA was used for normalization. β-actin was used as a loading control. **C**, **D** The levels of Col18a1, Endostatin, and Serpin E1 in βTC6 cells (**C**) or pancreatic tissues (**D**) were analyzed by immunofluorescence microscopy. The nuclei were stained with DAPI solution. Fluorescent signals between WT and HuD KO mice were quantified using the Image J program (**D**). Scale bar, 50 μm. Data indicate the mean ± SEM and images are representative of three independent experiments. The statistical significance of the data was analyzed via Student’s *t* test; *p < 0.05; **p < 0.01; ***p < 0.001.

To determine whether HuD affects the expression of Endostatin and Serpin E1 that are involved in the regulation of the growth and migration of vascular cells, the relative levels of mRNA and protein were assessed using RT-qPCR and western blotting analysis in βTC6 cells. Endostatin is a C-terminal fragment from the α chain of collagen XVIII (Col18a1) [30], so the expression of Col18a1 was measured instead of Endostatin. After HuD downregulation, there were no significant changes in the mRNA levels of *Col18a1* and *Serpin E1* (Fig. 3A). However, HuD knockdown increased the expression of Col18a1 and Serpin E1 in βTC6 cells (Fig. 3B). Furthermore, Col18a1/Endostatin and Serpin E1 levels assessed by western blotting analysis were also increased in the lysates and the medium of shHuD-βTC6 cells stably expressing small hairpin RNA (shRNA) against *HuD* [8] (Supplementary Fig. S2A). Additional study using immunofluorescence microscopy revealed that HuD downregulation increased the expression of Col18a1 and Serpin E1 in βTC6 cells, as well as pancreatic islet tissues (Figs. S2B and 3C, D). These results suggest that HuD downregulation increases the expression of Col18a1/Endostatin and Serpin E1.

**Fig. 4:**
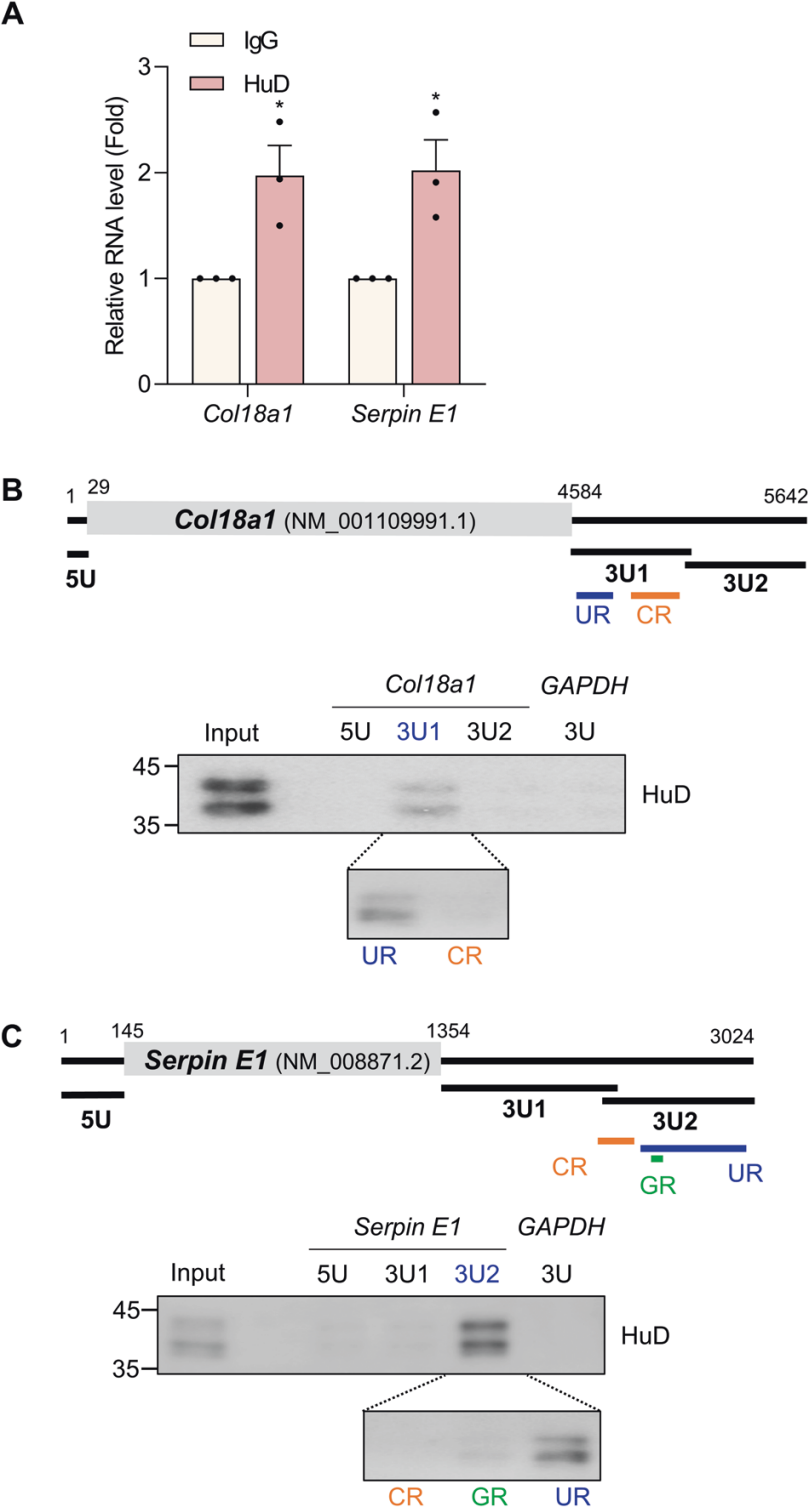
HuD interacts with *Col18a1* and *Serpin E1* mRNAs via their 3′UTRs. **A** HuD-containing ribonucleoprotein complexes were isolated via RNA immunoprecipitation and relative levels of *Col18a1* and *Serpin E1* mRNAs in IP complexes were analyzed by RT-qPCR. *Gapdh* mRNA was used for normalization. **B**, **C** Schematics of mouse *Col18a1* (NM_001109991.1) (**B**) and *Serpin E1* (NM_008871.2) (**C**). Each DNA fragment containing 5U, 3U1, 3U2, U-rich (UR), C-rich (CR), and G-rich (GR) regions of *Col18a1* and *Serpin E1* was transcribed in vitro using T7 RNA polymerase and biotin-labeled nucleotides. The biotinylated transcripts were incubated with βTC6 lysates, pulled down using streptavidin beads, and further assessed by western blotting analysis using HuD antibody. Biotinylated *GAPDH* 3U transcript was used as a negative control. Data indicate the mean ± SEM and images are representative of three independent experiments. The statistical significance of the data was analyzed via Student’s *t* test; *p < 0.05.

To elucidate the regulatory mechanism of HuD as an RNA binding protein, the association between HuD and mRNAs was assessed by RNA immunoprecipitation (RNA IP) analysis followed by RT-qPCR using the specific primers for *Col18a1* and *Serpin E1* mRNAs. Both *Col18a1* and *Serpin E1* mRNAs were found to be enriched in HuD IP (Fig. 4A), indicating that these mRNAs associate with the HuD-containing ribonucleoprotein complexes. To determine the HuD binding regions on *Col18a1* and *Serpin E1* mRNAs, a biotin-pulldown assay using biotin-labeled transcripts corresponding to the UTRs of *Col18a1* and *Serpin E1* mRNAs was performed. The binding between HuD and the transcripts was further assessed by western blotting analysis and the results showed that HuD bound to the U-rich regions in 3′UTR (3U-UR) of *Col18a1* mRNA of (4705–4800) nt and *Serpin E1* mRNA of (2401–2870) nt (Fig. 4B, C). These results suggest that HuD regulates *Col18a1* and *Serpin E1* expression by binding to those mRNAs.

**Fig. 5:**
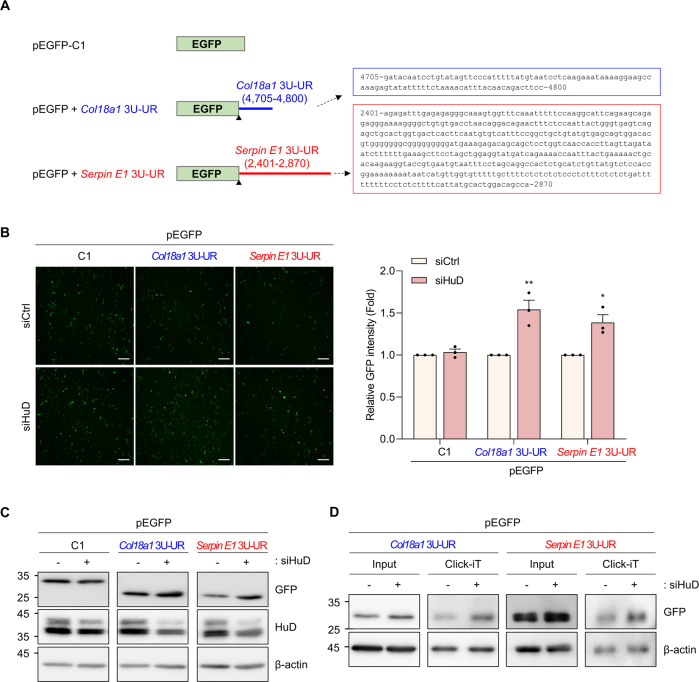
HuD knockdown promotes the expression of EGFP reporters containing 3′UTRs of *Col18a1* and *Serpin E1* mRNAs. **A** Schematics of EGFP reporters containing UR regions of *Col18a1* mRNA 3U (blue) and *Serpin E1* mRNA 3U (red). EGFP reporter plasmids, pEGFP + *Col18a1* 3U-UR and pEGFP + *Serpin E1* 3U-UR, were generated by inserting the UR regions of *Col18a1* (4,705-4,800 nt, blue rectangle) and *Serpin E1* (2,401-2,870 nt, red rectangle) mRNAs into pEGFP vector. ▲, termination codon. **B**, **C** After transfection of Neuro-2a cells with siRNAs and reporter plasmids, EGFP reporter expression was assessed by fluorescence microscopy (**B**) and western blotting analysis using GFP antibody (**C**). Scale bar, 200 μm. **D** Analysis of *de novo* protein synthesis using the Click-iT™ system. Newly synthesized proteins were metabolically labeled using L-azidohomoalanine (AHA) and biotin using the Click-iT™ reaction buffer. The biotin-labeled samples were isolated with streptavidin beads and assessed by western blotting analysis using GFP antibody. β-actin was used as a loading control. Data indicate the mean ± SEM and images are representative of three independent experiments. The statistical significance of the data was analyzed via Student’s *t* test; *p < 0.05; **p < 0.01.

The EGFP reporter assay was used to further confirm whether HuD regulates the expression of Col18a1 and Serpin E1 by interacting with their 3′UTRs. To generate EGFP reporter constructs containing HuD binding sites (pEGFP + *Col18a1* 3U-UR and pEGFP + *Serpin E1* 3U-UR), the (4705–4800) nt region of *Col18a1* mRNA 3′UTR or the (2401–2870) nt region of *Serpin E1* mRNA 3′UTR were inserted into pEGFP reporter plasmids behind the termination codon of EGFP, as shown in Fig. 5A. Fluorescence microscopy and western blotting analysis were used to examine EGFP levels after HuD knockdown. HuD downregulation increased the levels of EGFP containing 3U-UR regions of *Col18a1* and *Serpin E1* mRNAs, indicating that HuD negatively regulates Col18a1 and Serpin E1 expression by binding to their 3′UTR (Fig. 5B, C). Since there were no significant changes in mRNA level (Fig. 3A), *de novo* protein synthesis of the reporters was investigated using the Click-iT™ system to determine whether HuD affects the translation of *Col18a1* and *Serpin E1* mRNAs. HuD knockdown increased the levels of newly synthesized reporters (Fig. 5D), indicating that HuD knockdown promoted the translation of reporter constructs containing 3U-UR regions of *Col18a1* and *Serpin E1* mRNAs. These results suggest that HuD functions as a translational repressor in Col18a1 and Serpin E1 expression.

### Impaired cellular communications between β cells and islet endothelial cells by HuD knockdown

**Fig. 6:**
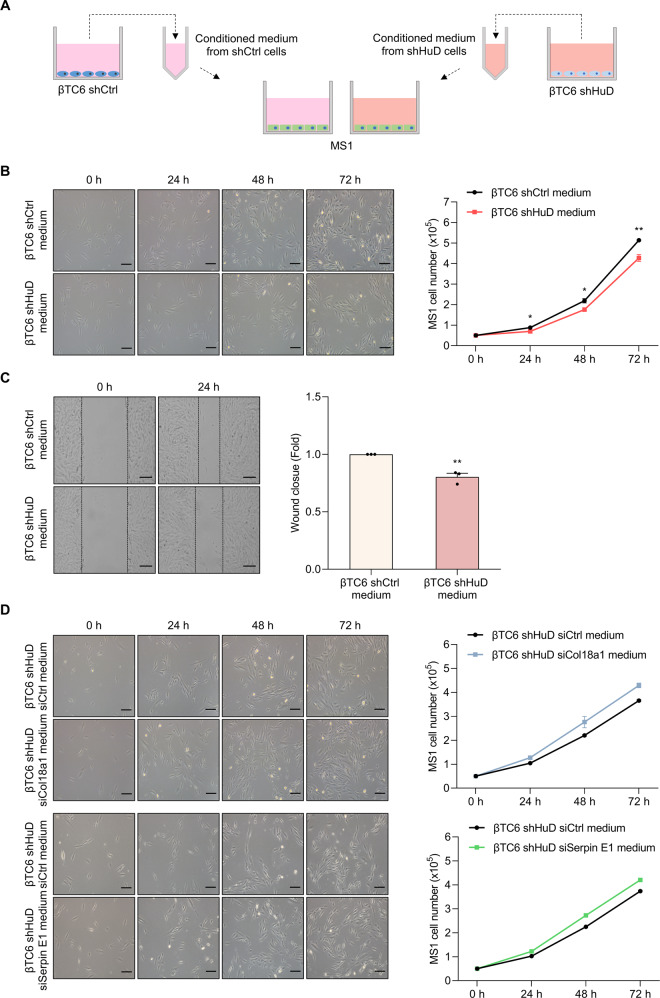
The conditioned medium from βTC6 shHuD cells decreases the growth and migration of MS1 cells. **A** Schematic diagram of the experimental procedure. **B**, **C** The conditioned medium from βTC6 shCtrl cells or βTC6 shHuD cells was collected and incubated with MS1 cells. The growth of MS1 cells was assessed by counting the number of cells at each time point (**B**). **C** Scratchwound healing assay. A scratch was made in the confluent monolayer culture of MS1 cells and cells were incubated with the conditioned medium of βTC6 cells. Relative wound closure of MS1 cells was quantified as the fold change of the migration distance to control distance after 24 h incubation. **D** After transfection of βTC6 shHuD cells with siRNAs, the conditioned medium was collected and incubated with MS1 cells. The growth of MS1 cells was assessed by counting the number of cells at each time point. Scale bar, 200 μm. Data indicate the mean ± SEM and images are representative. The statistical significance of the data was analyzed via Student’s *t* test; *p < 0.05; **p < 0.01.

Our findings showed the augmented expression of secretory proteins such as Col18a1/Endostatin and Serpin E1 by HuD knockdown in βTC6 cells. To see whether HuD can influence crosstalk between βTC6 cells and nearby cells by regulating the levels of secretory proteins, islet endothelial MS1 cells were cultured with the conditioned medium from βTC6 shCtrl cells and βTC6 shHuD cells, and then the growth and migration of MS1 cells were assessed by cell counting and scratch wound healing assay, as described in Fig. 6A. The conditioned medium from βTC6 shHuD cells decreased the number and migration of MS1 cells compared to βTC6 shCtrl cells (Fig. 6B, C). These results indicate that the conditioned medium from βTC6 shHuD cells reduced the growth and migration of MS1 cells. To further investigate whether HuD-mediated growth inhibition of MS1 cells is from the augmented expression of Endostatin and Serpin E1 in βTC6 shHuD cells, βTC6 shHuD cells were transiently transfected with siRNAs against *Col18a1* and *Serpin E1*, and the conditioned medium of βTC6 shHuD cells was incubated with MS1 cells. Cell counting analysis showed that *Col18a1* and *Serpin E1* knockdown in βTC6 shHuD cells restored the HuD-mediated growth inhibition of MS1 cells (Fig. 6D). These results suggest that βTC6 cells affect the growth and migration of MS1 cells by regulating Col18a1 and Serpin E1 expression. In addition, direct crosstalk between βTC6 cells and MS1 cells was assessed by co-culturing using transwell plates (Supplementary Fig. S3A). MS1 cells co-cultured with βTC6 shHuD cells had a lower number and reduced ability to close wounds than MS1 cells co-cultured with βTC6 shCtrl cells (Supplementary Fig. S3B, C). Taken together, these results suggest that HuD downregulation impairs the crosstalk between βTC6 cells and MS1 cells, thereby reducing the growth and migration of MS1 cells.

### Restoring HuD increased pericytes and endothelial cells in the islet microenvironment

**Fig. 7:**
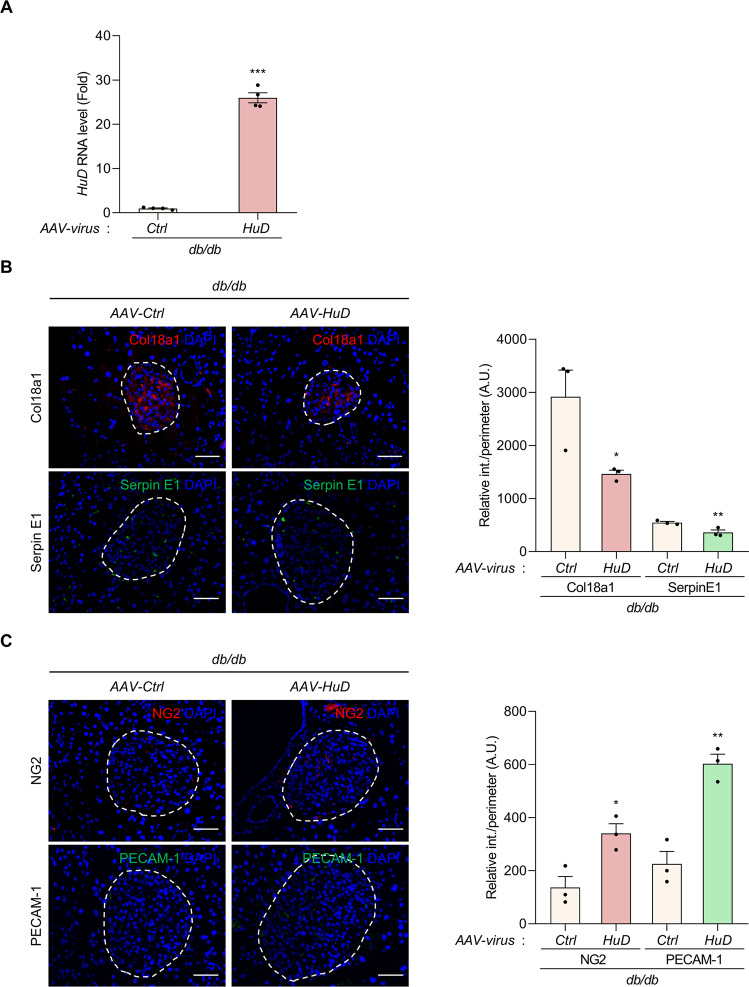
HuD overexpression decreases the levels of Col18a1 and Serpin E1, while increasing the markers of pericytes and endothelial cells. **A** *db/db* mice were infected with the AAV-HuD and AAV Blank control virus (AAV-Ctrl) (serotype 8) via the tail vein for 4 weeks. RNAs were isolated from the pancreas and relative expression of *HuD* mRNA was assessed by RT-qPCR. *Gapdh* mRNA was used for normalization. **B**, **C** Relative levels of Col18a1, Serpin E1, NG2, and PECAM-1 in the pancreatic tissues were analyzed by immunofluorescence microscopy. The nuclei were stained with DAPI solution. Fluorescent signals between AAV-Ctrl and AAV-HuD groups were quantified using the Image J program. Data indicate the mean ± SEM and images are representative of three independent experiments. Scale bar, 50 μm. The statistical significance of the data was analyzed via Student’s *t* test; *p < 0.05; **p < 0.01.

One of the animal models of type 2 diabetes mellitus, *db*/*db* mice have a lower level of HuD [7], while the number of islet vascular cells was reduced in diabetic islets [25]. To investigate the effect of HuD restoration in the islet microenvironment, HuD was overexpressed into the pancreas of *db*/*db* mice using the AAV system containing the *HuD* gene. RT-qPCR was used to verify HuD overexpression in the pancreas (Fig. 7A) and immunofluorescence microscopy was performed to determine the relative expressions of several proteins, including Col18a1, Serpin E1, NG2, and PECAM-1 (Fig. 7B, C). HuD overexpression significantly decreased Col18a1 and Serpin E1 expression in the pancreatic islet of *db*/*db* mice (Fig. 7B), while increasing the levels of marker proteins of pericytes and endothelial cells, NG2 and PECAM-1 (Fig. 7C). These results suggest that dysregulation of HuD is related to the impaired islet microenvironment and that HuD overexpression has the potential to restore the homeostasis of vascular cells in the diabetic islet.

## Discussion

Pancreatic islet contains hormone-releasing endocrine cells that govern glucose homeostasis, including α cell (glucagon), β cell (insulin), and δ cell (somatostatin) [31]. Recent studies assessed by single-cell RNA sequencing analysis have shown that the islet has a complex and highly ordered microenvironment that is composed of several endocrine cells, endothelial cells, pericytes, macrophages, neurons, and various types of ECM in human and mouse [32, 33]. Dysfunction of the islet is clinically important in pancreatitis, type 1 and 2 diabetes mellitus, and cancer; therefore, tight regulation of the islet microenvironment is essential for the intact integrity and function of the islet. Cells that live in the islet microenvironment communicate with each other by secreting soluble factors or extracellular vesicles (EVs), and by forming GAP junctions or nanotubes between adjacent cells [34]. The secretory activity of β cells leads to the proliferation and migration of nearby cells, including endothelial cells, pericytes, and pancreatic stellate cells, production of ECM proteins, and macrophage infiltration [21, 35]. Insulin, ATP, serotonin, VEGFA, and proinflammatory cytokines have been reported to be released from β cells and affect the islet microenvironment. Intra-islet endothelial cells regulate the proliferation and development of β cells by secreting connective tissue growth factor (CTGF) and thrombospondin-1 (TSP-1) [36–38]. Nerve growth factor (NGF) and bone morphogenetic protein 4 (BMP4) produced by pericytes also play essential roles in regulating β cell maturity and blood flow of the islet [24, 39]. In chronic inflammatory conditions, macrophages generate several inflammatory cytokines, such as interleukin-1β (IL-1β), and contribute to β cell dysfunction [29, 40]. Although several reports suggest the importance of the crosstalk between cells in the islet, further studies are needed to fully understand the dynamic and complex networks that contribute to the homeostasis of the islet microenvironment. In this study, we demonstrate that an RNA binding protein HuD stimulates crosstalk between β cells and islet endothelial cells by regulating the expression of Endostatin and Serpin E1 in pancreatic β cells. Our results suggest that HuD has the potential to regulate cell-to-cell communication by directing the level of soluble factors and that their aberrant expressions may lead to β cell dysfunction by impairing the homeostasis of the islet microenvironment.

In this study, we demonstrate that HuD-mediated alteration of secretory proteins in pancreatic β cells interfered with the homeostasis of the islet microenvironment. Since HuD is also expressed in neurons, HuD-mediated regulation that occurs in neurons found in the intra-islet may also affect the cellular communication within the islet. Our survey using Neuro-2a cells showed that some of the soluble factors involved in angiogenesis, including endostatin and fractalkine, were upregulated in the medium of HuD-knockdown neuronal cells (data not shown) [14]. This suggests that HuD plays an essential role in the regulation of dynamic and complex crosstalk among cells in the islet microenvironment. In addition, HuD is known to regulate various types of non-coding RNAs (ncRNAs), such as microRNAs, circular RNAs, and long non-coding RNAs [2, 5, 41], which implies the possibility that HuD mediates the crosstalk via ncRNA-containing extracellular vesicles, such as exosomes. Further investigation may allow understanding the comprehensive role of HuD in cell-to-cell communication involved in the maintenance of homeostasis in various tissues.

Reduction of HuD has been reported in the model of diabetes, and its aberrant level is involved in disease development, including type 2 diabetes mellitus, resulting in β cell dysfunction [7, 8]. We observed that the ectopic expression of HuD in the *db*/*db* mice restored the number of vascular cells in the islet (Fig. 7C), suggesting that the proper level of HuD contributes to maintaining the homeostasis of the islet microenvironment. It remains to fully elucidate why HuD is downregulated, or which mechanisms are involved in the downregulation of HuD during the pathogenesis of diabetes. However, restoring abnormal expression of HuD may be a strategy to improve the symptoms caused by β cell dysfunction. Additional studies on essential factors regulating HuD expression and their signaling pathways are necessary to fully understand the role of HuD in disease pathogenesis, including diabetes.

Several studies have shown that abnormal islet vascularization is associated with obesity and insulin resistance, resulting in inflammation, impaired insulin release, and β cell death [21, 36]. Both the thickening and fragmentation of islet capillaries and the expression of inflammation markers in endothelial cells have been reported in human and animal models of diabetes. However, some morphological and functional abnormalities in the islet microenvironment, such as capillary loss, hypertrophy of pericytes, and islet edema, are observed in rodent models of diabetes, but not in humans [42–44]. In this study, we demonstrated the HuD-mediated crosstalk between β cells (βTC6) and islet endothelial cells (MS1) using mouse cell lines. Further efforts need to determine whether observations from rodent models and in vitro culture systems are relevant to human diabetes and to fully elucidate the common mechanisms of the abnormal islet microenvironment that causes diabetes.

In summary, a growing body of evidence supports the concept that dysfunction of the islet microenvironment is a complex process, involving abnormal insulin secretion, impaired islet vascularization, immune cell infiltration, cytokine production, β cell death, and fibrosis, and contributes to the pathological process of various pancreatic-related diseases. We demonstrated that the crosstalk between β cells and islet endothelial cells is mediated by soluble factors that are released and regulated by HuD in β cells. These results suggest the potential role of HuD as a novel regulator to orchestrate the homeostasis of the islet microenvironment. Comprehensive elucidation of molecular mechanisms will provide an emerging target for the effective treatment and care of diabetes caused by dysfunction of the islet microenvironment.

## Materials and methods

### Cell culture and transfections

Mouse insulinoma βTC6, neuroblastoma Neuro-2a, and stable cells expressing short hairpin RNAs (shRNAs) (βTC6 shCtrl and βTC6 shHuD) [7] were cultured within Dulbecco’s modified Eagle’s medium (DMEM) (Capricorn Scientific, Ebsdorfergrund, Germany) supplemented with 10% fetal bovine serum (FBS) and 1% antibiotics, in the presence of 5% CO_2_. Pancreatic islet endothelial MS1 cells were cultured in DMEM/5% FBS/1% antibiotics. Enhanced green fluorescent protein (EGFP) reporter was generated by cloning the 3′UTR sequence of *Col18a1* or *Serpin E1* mRNA into the pEGFP-C1 (BD Bioscience, Franklin Lakes, NJ, USA) vector. EGFP reporter plasmids and small interfering RNAs (Genolution Pharmaceuticals, Inc., Seoul, South Korea) were transfected using Lipofectamine™ 2000 (Invitrogen™, Waltham, MA, USA), according to the manufacturer’s instructions.

### RNA analysis

Total RNAs were isolated from whole cells or mice tissues using RNAiso Plus (Takara Bio, Inc., Shiga, Japan). RNAs enriched in the HuD-containing ribonucleoprotein (RNP) complex were isolated by immunoprecipitation using Protein A bead (Invitrogen™) incubated with anti-HuD or control IgG antibody (Santa Cruz Biotechnology, Inc., Dallas, TX, USA) and extracted from the RNP complex by sequential incubation with DNase I and proteinase K. Complementary DNA (cDNA) was synthesized by reverse transcription using ReverTra Ace™ qPCR RT Kit (Toyobo Co., Ltd., Osaka, Japan) and quantitative PCR (qPCR) was performed using the SensiFAST™ SYBR Hi-ROX kit (Meridian Bioscience, Inc., Cincinnati, OH, USA), gene-specific primers (Supplementary Table S1), and StepOnePlus™ Real-Time PCR System (Applied Biosystems™, Waltham, MA, USA). Data were processed using the _ΔΔ_CT method for comparison between control and experimental groups.

### Western blotting analysis

Whole cell lysates were prepared using RIPA buffer (50 mM Tris-HCl (pH 7.5), 150 mM NaCl, 1% Triton X-100, 1% sodium deoxycholate, 0.1% SDS, and 2 mM EDTA) containing 1× protease inhibitor cocktail (Roche, Basel, Switzerland). The samples were mixed with SDS sample buffer, separated by SDS-polyacrylamide gel electrophoresis (SDS-PAGE), and transferred onto polyvinylidene difluoride (PVDF) membranes (Millipore, Burlington, MA, USA). The membranes were incubated with primary antibodies including HuD, GFP (Santa Cruz Biotechnology, Inc.), Col18a1 (Invitrogen™), Serpin E1 (Abcam Plc., Cambridge, UK), and β-actin (Genetex, Inc., Irvine, CA, USA) at 4 °C overnight, and further incubated with horseradish peroxidase (HRP)-conjugated secondary antibodies (Sigma-Aldrich, Burlington, MA, USA). Chemiluminescence was detected with the Clarity Western ECL Substrate (Bio-Rad, Inc., Hercules, CA, USA) using the ChemiDoc Imaging Systems (Bio-Rad, Inc.).

For the analysis of de novo protein synthesis, the Click-iT™ system (Invitrogen™) was used according to the manufacturer’s instructions. In brief, cells were sequentially transfected with siRNAs and EGFP reporters for 48 h and incubated in a methionine-free medium for 1 h. The medium was replaced with an AHA-containing medium for 4 h, and newly synthesized proteins were metabolically labeled with biotin using the Click-iT™ reaction buffer. The biotin-labeled samples were isolated with streptavidin-coupled Dynabeads (Invitrogen™), and further investigated by western blotting analysis using GFP antibody. Uncropped western blots were uploaded as a supplementary file.

### Cytokine array

After transfection of βTC6 cells with siRNAs or plasmids, a conditioned medium from each cell culture was collected to analyze secretory proteins using the Proteome Profiler Mouse XL Cytokine Array Kit (R&D Systems, Inc., Minneapolis, MN, USA) according to the manufacturer’s instructions. In brief, the conditioned medium from βTC6 cells was concentrated by centrifugation using Amicon® Ultra centrifugal filter (Millipore), followed by incubation with 111 different anti-mouse cytokine antibodies spotted on the Mouse XL Cytokine Array nitrocellulose membranes. Captured proteins were further incubated with detection antibodies and visualized using chemiluminescent detection reagents [14].

### Biotin pull-down assay

DNA fragments corresponding to the 3′UTRs of *Col18a1* and *Serpin E1* mRNAs were amplified by PCR using forward primers including T7 RNA polymerase binding sequence (5′-CCAAGCTTCTAATACGACTCACTATAGGGAGA-3′) [8]. Supplementary Table S1 lists the primers used for the PCR of *Col18a1* and *Serpin E1* mRNAs. After purification of the PCR products, biotinylated transcripts were synthesized using the MaxiScript T7 kit (Ambion, Waltham, MA, USA) and biotin-CTP (Enzo Life Sciences, Farmingdale, NY, USA). Whole cell lysates were incubated with purified biotinylated transcripts for 30 min at room temperature. The complexes of biotinylated transcripts and proteins were isolated using streptavidin-coupled Dynabeads (Invitrogen™), and further studied by western blotting analysis using HuD antibody.

### Immunofluorescence microscopy

Cells were fixed with 4% FA solutions and paraffin-embedded pancreatic tissues were deparaffinized. Fixed cells and tissues were permeabilized with Triton X-100, sequentially incubated with blocking solution and primary antibodies, including Col18a1 (Invitrogen™), Serpin E1, CD68 (Abcam Plc.), NG2 (Sigma-Aldrich), PECAM-1 (Santa Cruz Biotechnology, Inc.), and MPO (Agilent Technologies, lnc., Santa Clara, CA, USA), at 4 °C overnight, and further incubated with secondary antibodies conjugated with Alexa Flour® 488 or Alexa Flour® 555 (Abcam Plc.). DAPI (4’,6-diamidino-2-phenylindole) solution (Invitrogen™) was used to stain the nuclei. Fluorescence signals were observed and imaged using the ZEISS Axio Imager M1 microscope (Carl Zeiss, Oberkochen, Germany).

### Co-culture and analysis of the growth and migration of MS1 cells

βTC6 cells and MS1 cells were co-cultured using Falcon® Cell culture inserts and their companion plates (Corning Inc., NY, USA). βTC6 cells were grown in Falcon® Permeable Support and MS1 cells were seeded in the lower chamber of the plate. The number of MS1 cells was determined by cell counting with a hemocytometer under Leica DM IL LED microscope (Leica Microsystems Ltd, Wetzlar, Germany). For wound healing assay, the scratch was made in monolayer culture of MS1 cells using a 200-μL pipette tip, and the wound closure was analyzed using a Leica DM IL LED microscope (Leica Microsystems Ltd) 24 h later.

### Injection of AAV into animals

The study protocol using animals was approved by the Institutional Animal Care and Use Committee (IACUC) of the College of Medicine, Catholic University of Korea. AAV-HuD and AAV Blank control virus (serotype 8) were purchased from Applied Biological Materials Inc. (Richmond, BC, Canada). In all, 5 × 10^8^ genome copies (GC) of the virus were injected via the tail vein of C57BL/KsJ-*db/db* mice (12 weeks, *n* = 5). Four weeks later, the mice were sacrificed, and the pancreas was isolated for analysis.

### Statistical analysis

Data were expressed as the mean ± SEM of three independent experiments. The statistical significance of the data was analyzed via Student’s *t* test (*p < 0.05; **p < 0.01; ***p < 0.001).

## Supplementary information

Uncropped Western blot images

Reproduciblility checklist

Supplementary materials

## Data Availability

The data used and analyzed during the current study are available within the manuscript and its additional files. Additional data are available from the corresponding author upon reasonable request.
