# Supplementary figures and images for: RNA binding protein HuD mediates the crosstalk between β cells and islet endothelial cells by the regulation of Endostatin and Serpin E1 expression
Source: Cell Death Dis. 2022 Dec 5;13(12):1019. doi: 10.1038/s41419-022-05465-6 (PMC9722926; doi:10.1038/s41419-022-05465-6)

**Fig 1A and C**

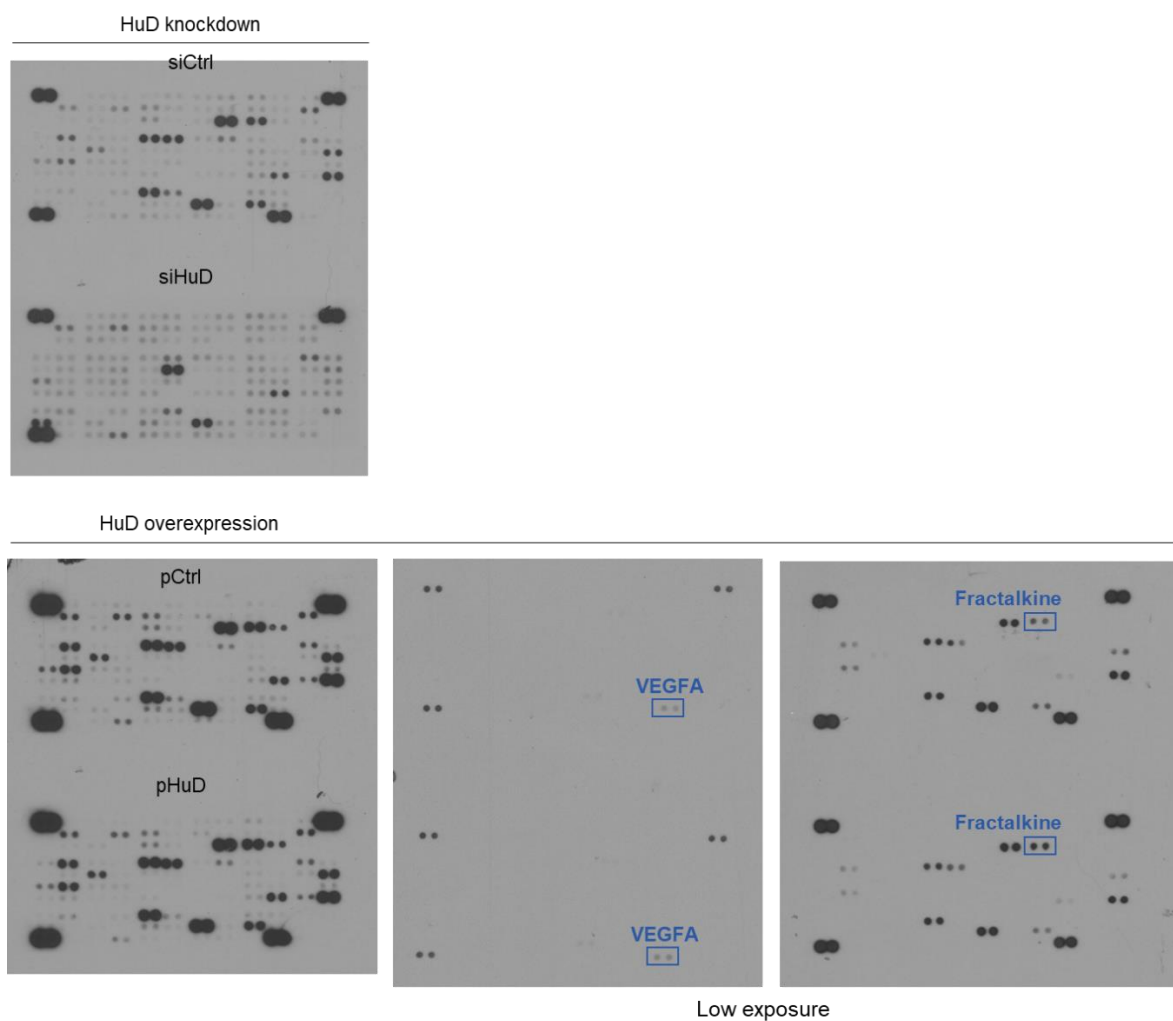

**Fig 1D**

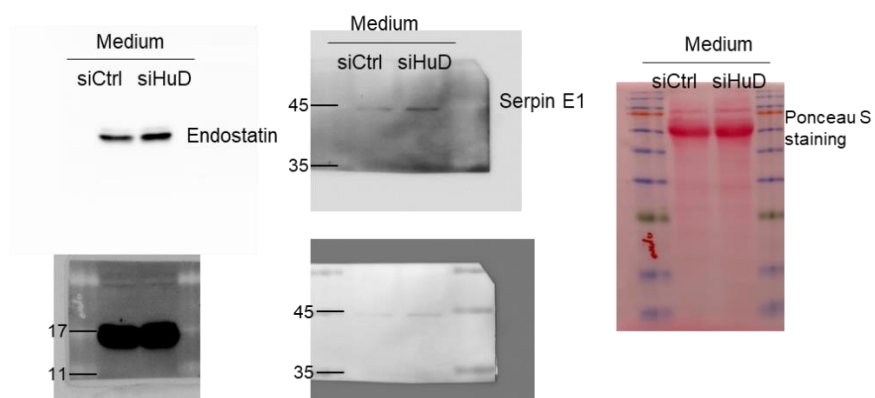

Fig 3B

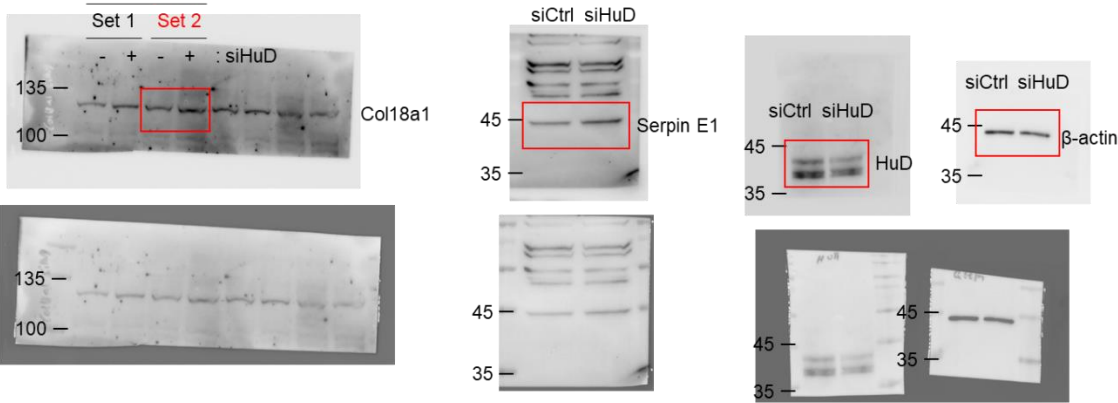

Fig 4B

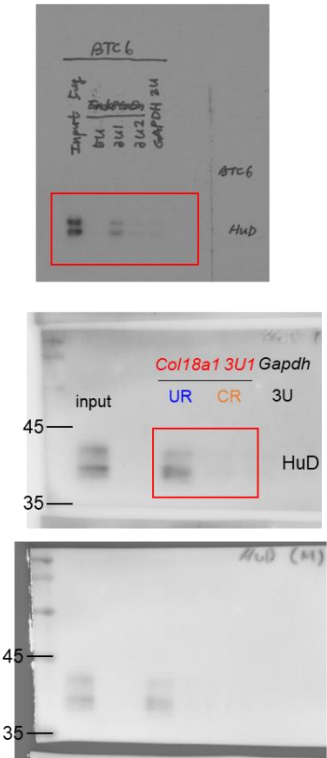

Fig 4C

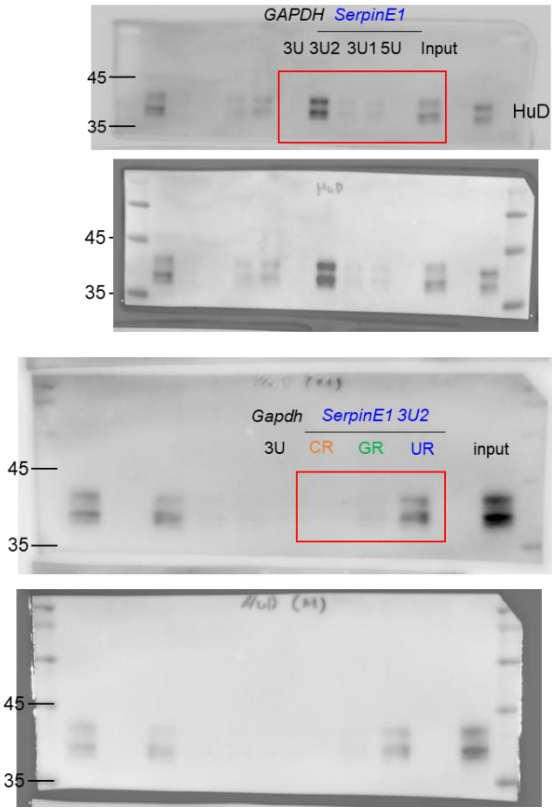

**Fig 5C**

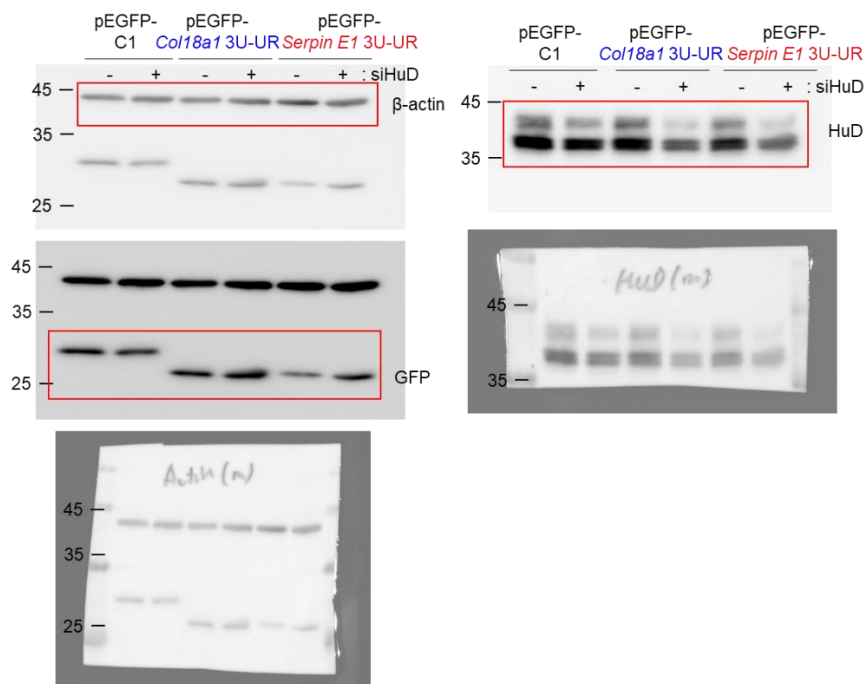

**Fig 5D**

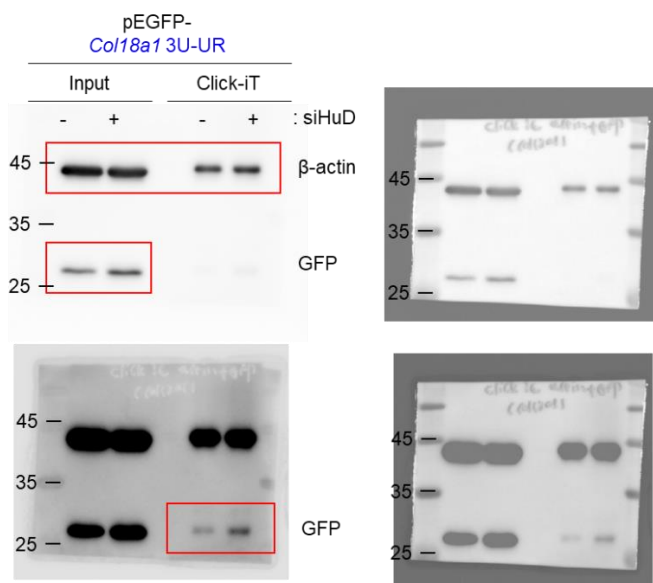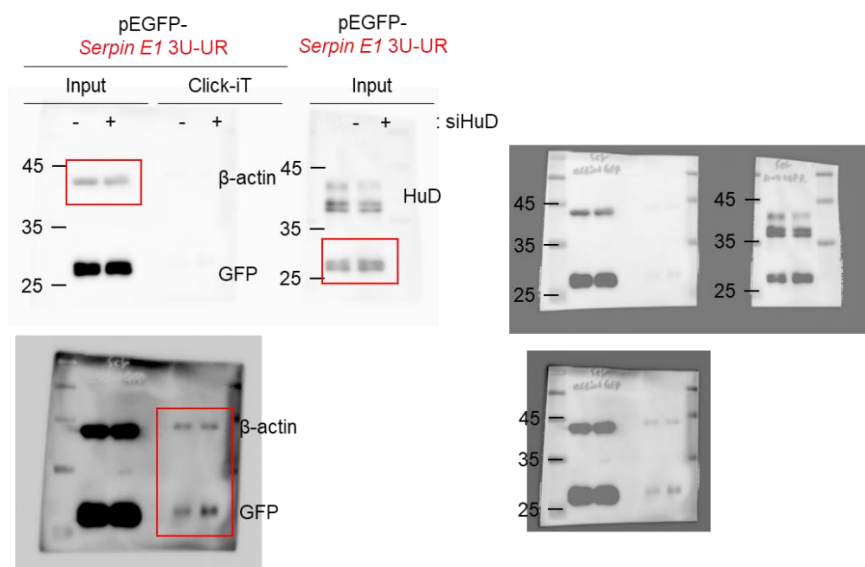

**Fig S1**

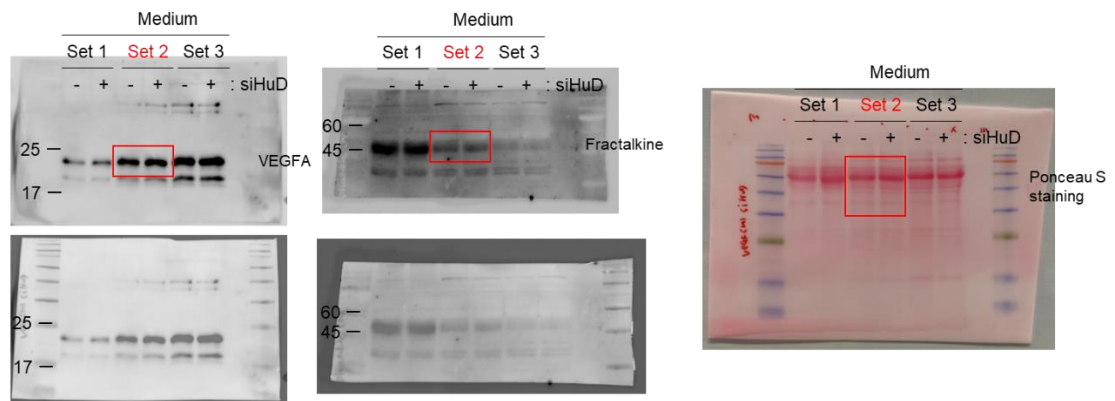

**Fig S2**

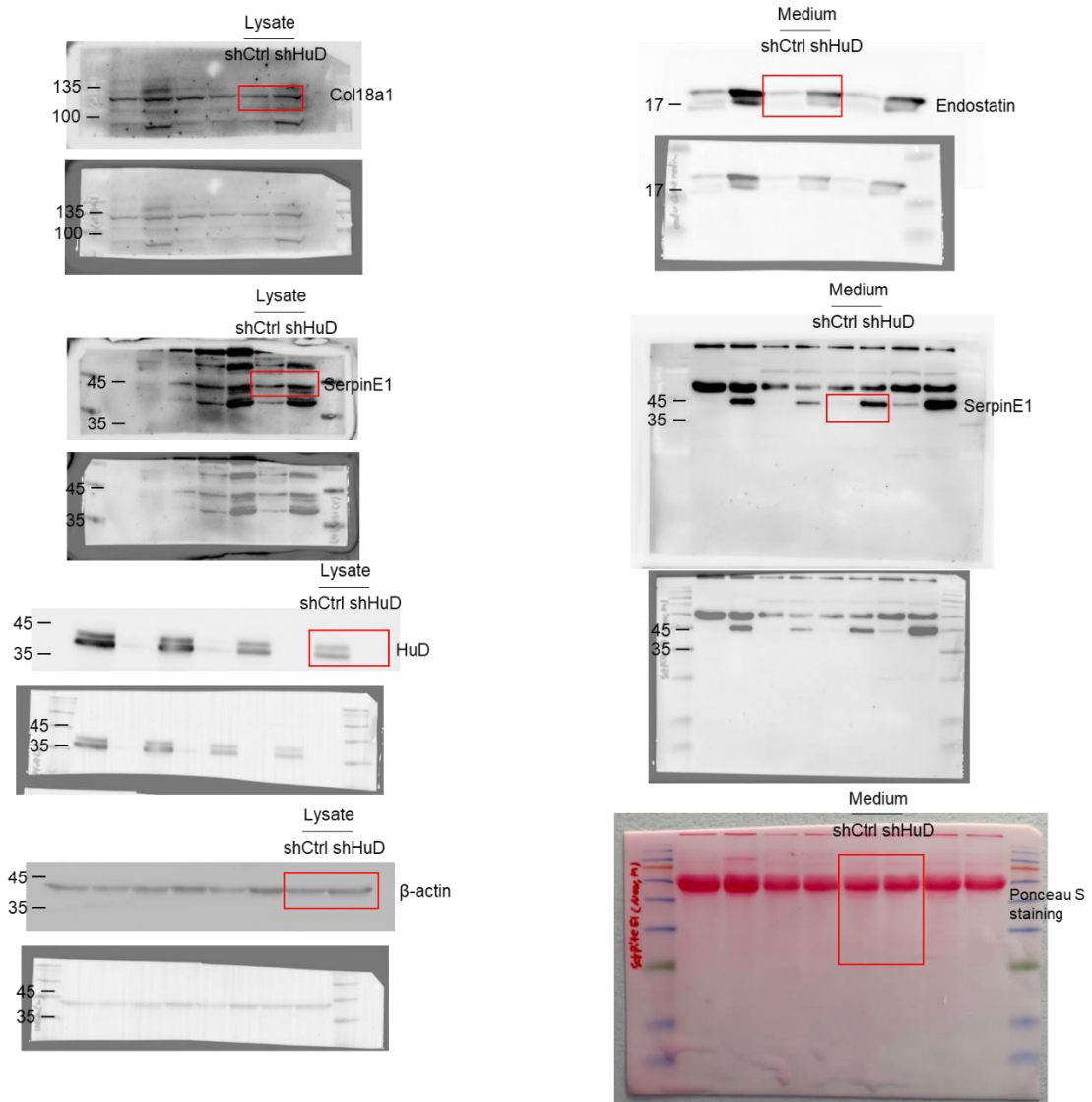

Supplement: Supplementary file 1 — Uncropped Western blot images [file 41419_2022_5465_MOESM1_ESM.pdf]
