# Supplementary material for: RNA binding protein HuD mediates the crosstalk between β cells and islet endothelial cells by the regulation of Endostatin and Serpin E1 expression
Source: Cell Death Dis. 2022 Dec 5;13(12):1019. doi: 10.1038/s41419-022-05465-6 (PMC9722926; doi:10.1038/s41419-022-05465-6)
Supplement: Supplementary file 3 — Supplementary materials [file 41419_2022_5465_MOESM3_ESM.docx]

**RNA binding protein HuD mediates the crosstalk between β cells and islet endothelial cells by the regulation of Endostatin and Serpin E1 expression.**

​Myeongwoo Jung^1, 2^, Seungyeon Ryu^1, 2^, Chongtae Kim^2, 3^, Seongho Cha^1, 2^, Hoin Kang^2, †^, Eunbyul Ji^2^, Youlim Hong^2^, Youngjoon Lee^1, 2^, Sukyoung Han^1, 2^, Seung Min Jeong^1, 2, 4^, Wook Kim^5^, and Eun Kyung Lee^1, 2, 4^ *

^1^ Department of Biomedicine & Health Sciences, ^2^ Department of Biochemistry, ^3^ Catholic Institute for Visual Science, ^4^ Institute for Aging and Metabolic Diseases, College of Medicine, The Catholic University of Korea, Seoul 06591, South Korea

^5^ Department of Molecular Science & Technology, Ajou University, Suwon 16499, South Korea.

^†^ Current address: Jeju Institute of Korean Medicine, Jeju 63309, South Korea.

* Correspondence should be addressed to Eun Kyung Lee; [leeek@catholic.ac.kr](mailto:leeek@catholic.ac.kr)

**Supplementary Table S1. The oligonucleotide sequences used in this study**

| **For qPCR** | **Forward primer (5’ → 3’)** | **Reverse primer (5’ → 3’)** |
| --- | --- | --- |
| *Col18a1* | TCTATAGGAGCTGAGACCCTTC | TGTCCTTTCATACCTCCCTTTATC |
| *Serpin E1* | CAAGCTCTTCCAGACTATGGTG | ACCTTTGGTATGCCTTTCCAC |
| *HuD* | GCCTCAGGTGTCAAATGGACC | CCATACCCTAAACTCTGTCCTGT |
| *Gapdh* | AGGTCGGTGTGAACGGATTTG | TGTAGACCATGTAGTTGAGGTCA |
| **For BPD** | **T7 + Forward primer (5’ → 3’)**  (T7: CCAAGCTTCTAATACGACTCACTATAGGGAGA) | **Reverse primer (5’ → 3’)** |
| *Col18a1* 5U | (T7) GCCCAGCGCAGAGGCTCTCACTGCCCTG | CAGGGCAGTGAGAGCCTCTGCGCTGGGCTCTCCCTATAGTGAGTCGTATTAGAAGCTTGG |
| *Col18a1* 3U1 | (T7) GGCCTCTGCCAGCTAGGG | GCAGGAGAGTGGGGCAGTC |
| *Col18a1* 3U2 | (T7) GGTAACTGGGTCTACTCATCC | GGTATAAAATGTCATTTTTATTTGGTG |
| *Col18a1* 3U-UR | (T7) GATACAATCCTGTATAGTTCCC | GGAAGTCTGTTGTAAATGTTT |
| *Col18a1* 3U-CR | (T7) AACAGACTTCCTGCCCTTCCTC | AGCCACTGGGCGGTGAGAA |
| *Serpin E1* 5U | (T7) GAGCACAGCTGGATCAGG | CCTGAGTATTCAAAGGTGCCT |
| *Serpin E1* 3U1 | (T7) CAGTGGGAAGAGACGCCTTCA | GCAGGTTTGTGGAGTGATGC |
| *Serpin E1* 3U2 | (T7) CTGTATGTCAGGGGTGCATC | GGCATATGTAACCAACAAAATTC |
| *Serpin E1* 3U-CR | (T7) GTGCATCACTCCACAAACCTG | CAAGGTCCCTAAGGAAAAGAG |
| *Serpin E1* 3U-GR | (T7) CGTGGGGGGGCGGGGGGGGGAT | ATCCCCCCCCCGCCCCCCCACGTCTCCCTATAGTGAGTCGTATTAGAAGCTTGG |
| *Serpin E1* 3U-UR | (T7) AGAGATTTGAGAGAGGGCAAAG | TGGCTGTCCAGTGCATAATG |
| *GAPDH* 3U | (T7) GAGACCTCAACGACCACTTTGTCA | GGTTGAGCACAGGGTACTTTAT |
| **For cloning** | **Forward primer (5’ → 3’)** | **Reverse primer (5’ → 3’)** |
| *Col18a1* 3U-UR | AAAAAGATCTTAAGATACAATCCTGTATAGTTCCC | AAAAGGTACCGGAAGTCTGTTGTAAATGTTT |
| *Serpin E1* 3U-UR | AAAAAGATCTTAAAGAGATTTGAGAGAGGGCAAAG | AAAAGGATCCTGGCTGTCCAGTGCATAATG |
| **siRNA** | **Sense sequence (5’ → 3’)** | **Antisense sequence (5’ → 3’)** |
| siControl (siCtrl) | AAUUCUCCGAACGUGUCACGUUU | ACGUGACACGUUCGGAGAAUUU |
| siHuD #1 | GAAUGAACAUCCCUGGUCAUU | UGACCAGGGAUGUUCAUUCUU |
| siHuD #2 | CCUCAUCGUCAACUAUUUAUU | UAAAUAGUUGACHAUGAGGUU |
| siHuD #3 | GACAGAGUGUUGCAAGUUUUU | AAACUUGCAACACUCUGUCUU |
| siHuD #4 | GAGUUUAGGGUAUGGAUUUUU | AAAUCCAUACCCUAAACUCUU |
| siCol18a1 | CUCUAUGUACGCGUUAGAAUU | UUCUAACGCGUACAUAGAGUU |
| siSerpin E1 | CAAUGGAAGGGCAACAUGAUU | UCAUGUUGCCCUUCCAUUGUU |

**Supplementary Table S2. The source of antibodies used in this study**

| **Antibody** | **Company** | **Catalog number** |
| --- | --- | --- |
| HuD | Santa Cruz Biotechnology, Inc. | sc-28299 |
| β-actin | GeneTex, Inc. | GTX629630 |
| Col18a1/Endostatin | Invitrogen™ | PA1-601 |
| Serpin E1 | Abcam Plc. | ab66705 |
|  |  | ab125687 |
| NG2 | Sigma-Aldrich | AB5320 |
| PECAM-1 | Santa Cruz Biotechnology, Inc. | sc-376764 |
| MPO | Agilent Technologies, Inc. | A0398 |
| CD68 | Abcam Plc. | ab31630 |
| GFP | Santa Cruz Biotechnology, Inc. | sc-9996 |
| Mouse IgG (HRP Conjugated) | Sigma-Aldrich | AP124P |
| Rabbit IgG (HRP Conjugated) | Sigma-Aldrich | AP132P |
| Mouse IgG H&L (Alexa Fluor® 488) | Abcam Plc. | ab150113 |
| Rabbit IgG H&L (Alexa Fluor® 555) | Abcam Plc. | ab150074 |
| Normal mouse IgG | Santa Cruz Biotechnology, Inc. | sc-2025 |


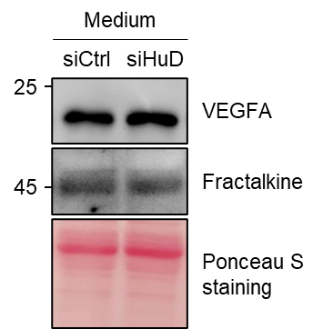


**Supplementary Figure S1. Relative levels of VEGFA and Fractalkine in the conditioned medium of βTC6 cells**

After transfection of βTC6 cells with siRNAs, the levels of VEGFA and Fractalkine in the conditioned medium were assessed by western blotting analysis using VEGFA and Fractalkine antibodies. Ponceau S staining was used for total protein normalization. Images are representative of three independent experiments.


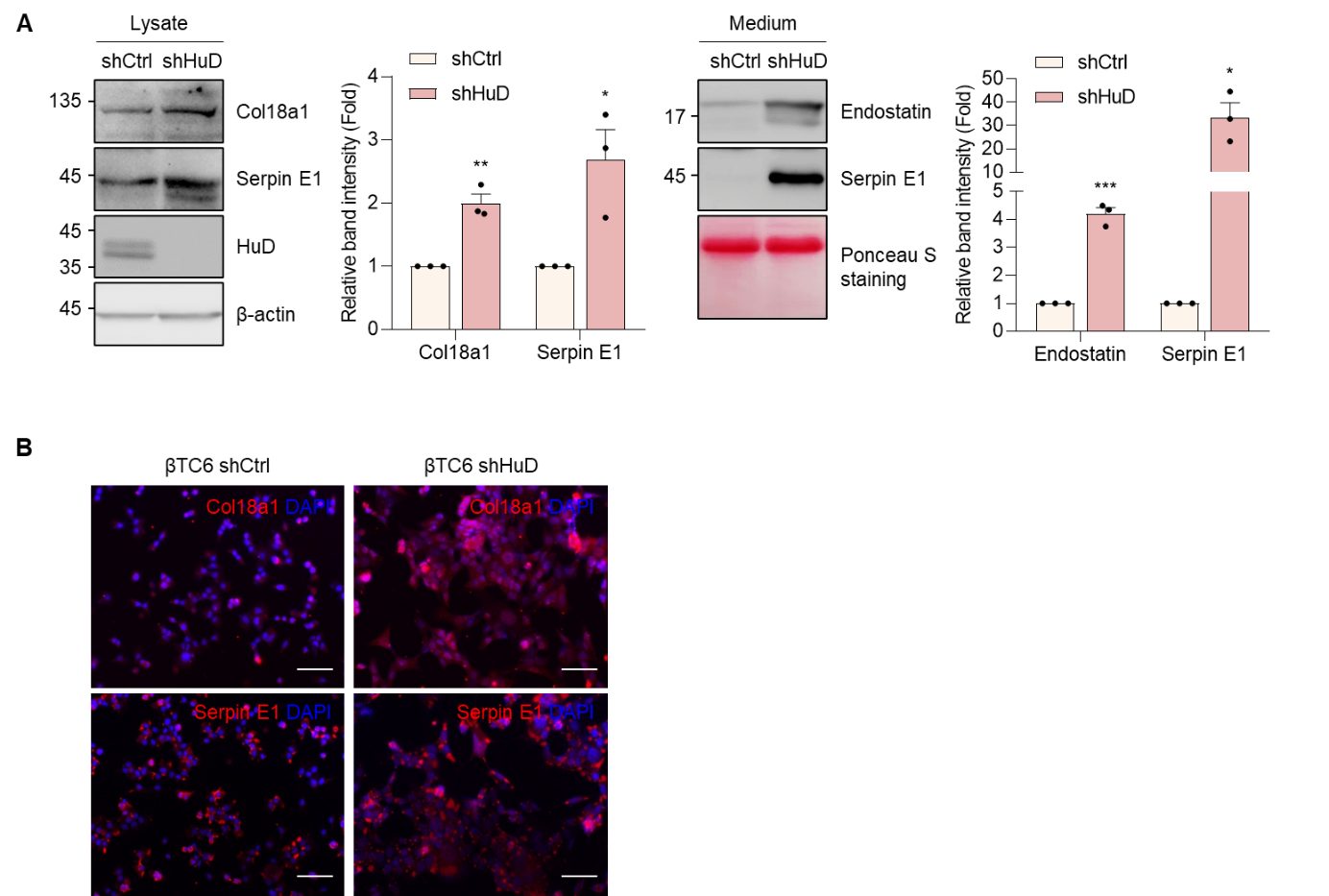


**Supplementary Figure S2. Augmented expression of Col18a1/Endostatin and Serpin E1 in βTC6 shHuD cells**

The levels of Col18a1, Endostatin, and Serpin E1 between βTC6 shCtrl cells and βTC6 shHuD cells were determined by western blotting analysis (A) or immunofluorescence microscopy (B). β-actin was used as a loading control. Ponceau S staining was used for total protein normalization. The nuclei were stained with DAPI solution. Scale bar, 50 μm. Data indicate the mean ± SEM and images are representative from three independent experiments. The statistical significance of the data was analyzed via Student’s t-test; *, p < 0.05; **, p < 0.01; ***, p < 0.001.


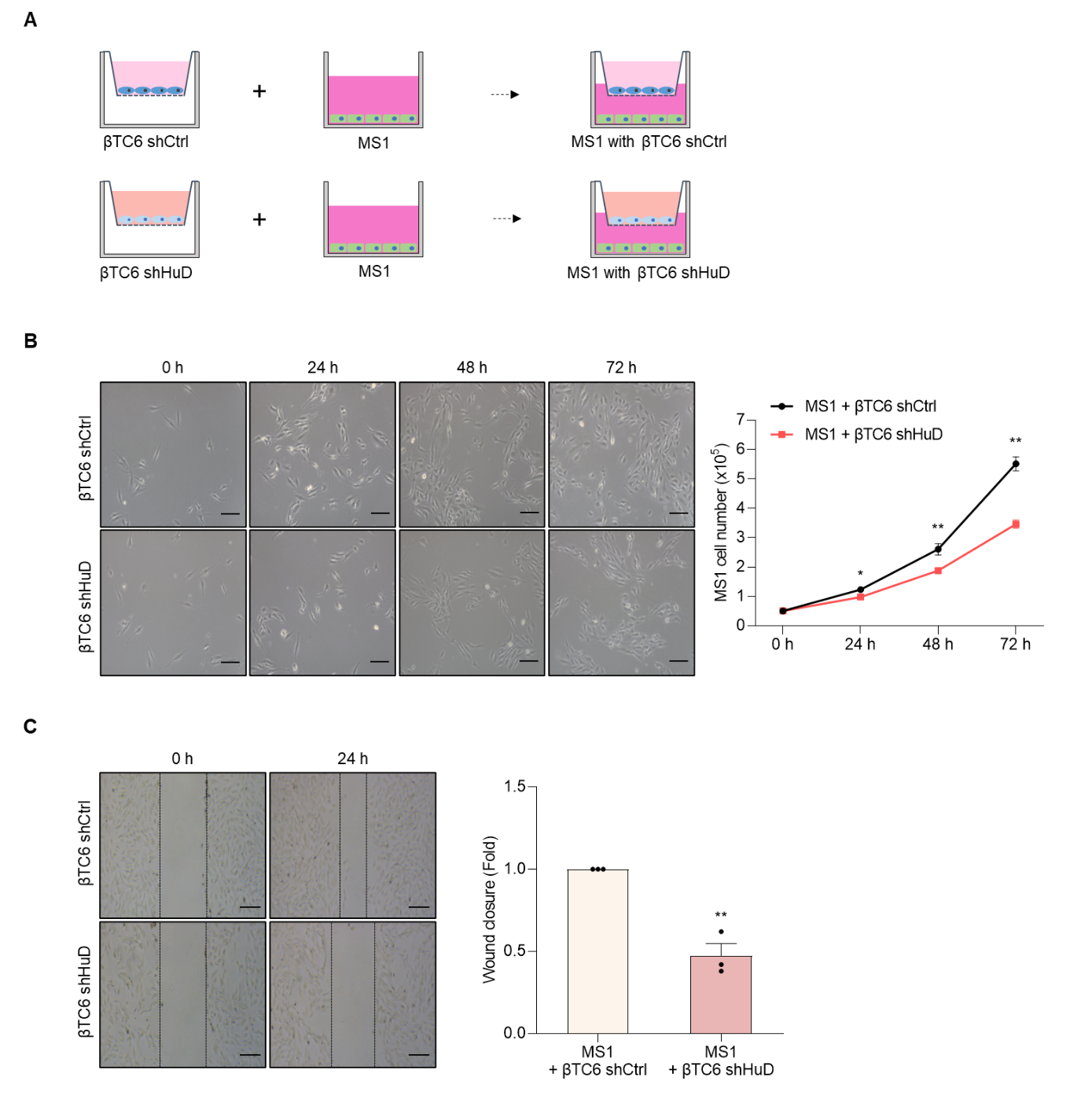


**Supplementary Figure S3. HuD downregulation in βTC6 cells decreases the growth and migration of MS1 cells.**

(A) Schematic diagram of the experimental procedure. (B and C) MS1 cells (lower chamber) were co-cultured with βTC6 shCtrl cells or βTC6 shHuD cells (upper chambers) using the transwell system. The growth of MS1 cells was assessed by counting the number of cells at each time point (B). (C) Scratch-wound healing assay. A scratch was made in the confluent monolayer culture of MS1 cells and the relative wound closure of MS1 cells was quantified as the fold change of the migration distance to control distance after 24 h incubation with βTC6 cells. Scale bar, 200 μm. Data indicate the mean ± SEM and images are representative from three independent experiments. The statistical significance of the data was analyzed via Student’s t-test; *, p < 0.05; **, p < 0.01.
